# Effects of tamoxifen on urinary incontinence: Case report and review of literature

**DOI:** 10.1097/MD.0000000000006785

**Published:** 2017-08-25

**Authors:** Elshad Hasanov, Merve Hasanov, Issa M. Kuria, Rovshan Hasanov, Reshad Rzazade, Eric Jonasch, Kadri Altundag

**Affiliations:** a Department of Medical Oncology, Hacettepe University Cancer Institute; b Department of Endocrinology and Metabolism, Hacettepe University School of Medicine, Ankara, Turkey; c Department of Genitourinary Medical Oncology, the University of Texas MD Anderson Cancer Center, Houston, TX, USA.

**Keywords:** breast cancer, tamoxifen, urinary incontinence

## Abstract

**Rationale:**

Tamoxifen has been used in women with hormone receptor-positive breast cancer and has been shown to successfully reduce both recurrence and mortality. On the contrary, long-term use of tamoxifen has hormone-related urogenital side effects which decrease the quality of life of the patients.

**Patient concerns:**

In this case report, we present a breast cancer patient receiving tamoxifen who developed urinary incontinence; we discuss the effects of tamoxifen on urinary incontinence, which decreases quality of life of the patients who were evaluated in our clinic.

**Diagnoses:**

Breast cancer, urinary incontinence.

**Interventions:**

Temporarily discontinuing tamoxifen.

**Outcomes:**

Urinary incontinence resolved.

**Lessons:**

Based on the case we reported and literature, estrogen can cause a dose-dependent increase in incontinence, but more preclinical and clinical studies of both estrogen and SERMs are needed to support this notion; given the fact that some small-scale clinical studies have not proven a direct relationship between tamoxifen and urinary incontinence. We suggest that clinicians faced with the issue should temporarily stop usage of the drug once the complaint of urinary incontinence arises.

## 1 Introduction

Breast cancer is an important cause of death. The use of tamoxifen in women with hormone receptor-positive breast cancer has been shown to reduce the recurrence by 41% and its mortality by 34% in comparison with placebo.^[1]^ Unfortunately, hormone-related side effects such as urogenital symptoms due to long-term use of this drug are inevitable, and this affects the quality of life.^[2]^ However, it should be noted that most of the frequently seen urogenital symptoms observed in healthy postmenopausal women are strongly linked to low endogenous estrogen levels observed in these women.^[3]^ Therefore, the long-term effect of hormonal therapy in postmenopausal women who are already predisposed to have urogenital complaints is a matter of debate. In this case report, we discuss the effects of tamoxifen on urinary incontinence, which has detrimental consequences on the quality of life of the patients who were admitted to our clinic. Ethics committee approved the study.

## 2 Case report

A 67-year-old postmenopausal female patient was found to have a mass in the upper outer quadrant of her left breast a year ago. She was diagnosed with ductal carcinoma in situ (DCIS) after surgical biopsy (T1NXMO). Following the diagnosis, a breast-conserving surgery was done. The tissue sent for pathological analysis was reported to be estrogen receptor positive (ER+), progesterone receptor negative (PR−), and grade II DCIS. Median pathologic tumor size was 8 mm. She received radiotherapy to the chest wall following the surgery. After the radiotherapy, 5-year adjuvant hormone therapy with tamoxifen was planned. However, after 4 months of tamoxifen treatment, the patient came to the clinic with complaints of urinary incontinence. Assessments with 2 standardized questionnaires, the Incontinence Impact Questionnaire (IIQ-7) and the Urogenital Distress Inventory (UDI-7), revealed a severe incontinence.^[4]^ Her thorough gynecological examination, which was performed by a gynecologist, was reported to be normal. It was therefore thought that the urinary incontinence was due to the use of tamoxifen and the drug was stopped. Two weeks later the patient visited the clinic and stated that the complaints had stopped and the IIQ-7/UDI-7 assessments confirmed her statement. Prior to the diagnosis of breast cancer, the patient had entered menopause at the age of 52 after a total abdominal hysterectomy-bilateral salpingo-oophorectomy performed for the treatment of myoma uteri. After the procedure, the patient underwent hormone replacement therapy for 96 months, and urinary incontinence arose similarly, which also resolved after the treatment was stopped.

## 3 Discussion

In one of the studies, which aimed to assess the rate of collagen type III synthesis by pubocervical fascia fibroblasts cultured with polypropylene meshes in the presence of estrogens and tamoxifen, the fibroblasts were obtained from pubocervical fascia sampled from a 52-year-old premenopausal woman who underwent surgical treatment for stress urinary incontinence (SUI). The fibroblasts were then cultured with monofilament or multifilament polypropylene meshes in the presence of 17B-estradiol, estriol, daidzein, or tamoxifen. N-terminal propeptide of type III procollagen (PIIINP) was used as a marker of collagen type III synthesis. The results showed that whereas the highest rate of collagen type III synthesis was observed in the culture treated with estriol, the highest total production of PIIINP was observed in culture treated with tamoxifen and it was thus concluded that the rate and/or production of collagen type III synthesis is subject to modulation by estrogens and antiestrogens.^[5]^ In this context, as a result of tamoxifen treatment, the increase in collagen production in extracellular matrix can support the urogenital organs and thus may reduce urinary incontinence.

In another study that was performed to evaluate the effects of tamoxifen on the weight and thickness of the urethral epithelium of castrated female rats, 40 rats were divided into 2 groups in which group 1 received propylene glycol and group 2 was given tamoxifen 250 μg/d. After 30 days of treatment, the urethral weight and thickness of the rats were measured. It was observed that there was a significant increase in the mean weight and the mean thickness of the urethra in the rats treated with tamoxifen in comparison with the control group (*P* < .001).^[6]^ A similar study was done to evaluate the effects of raloxifene on the weight and epithelial thickness of the urethra of castrated female rats. A total of 40 castrated female rats were randomly separated 2 two groups: group I (n = 20) was the control group whereas group II (raloxifene, n = 20) received 750 μg/d of raloxifene, for 30 days. At the end of the study, it was observed that raloxifene increased the distal urethral epithelial thickness (*P* < .05) but did not alter the weight of the urethra (*P* = .371) and the proximal urethral epithelial thickness (*P* = .187).^[7]^ Thus, treatment with tamoxifen may increase the weight of the urethra and the thickness of the urethral epithelium, which may reduce incontinence. Similarly, treatment with raloxifene may also reduce incontinence by increasing the distal urethral epithelial thickness mechanism.

In another study, application of estrogen, raloxifene, and levormeloxifene to isolated urethral smooth muscle cells was shown to have a dose-dependent reduction in the expression of RhoA, Rock-I, Rock-II, and p-MLC molecules, which are Rho-kinase signal transduction molecules.^[8]^ The Rho-kinase signaling pathway has a key role in controlling the contraction mechanisms of many smooth muscle cells.^[9]^ For this reason, it can be postulated that estrogen, raloxifene, and levormeloxifene cause urinary incontinence by reducing urethral resistance.

Last but not least, a study was done to evaluate in vivo modulation of the urinary bladder wall by estradiol and raloxifene in a rat model. A total of 30 castrated (ovariectomy) female rats were divided into 3 groups and fed either an estradiol-, raloxifene-, or unsupplemented soy-free formula for 10 weeks. Then, the urinary bladder was filled via a transurethral catheter for recording the intravesical pressure during a stretch period and a 1 min isometric accommodation period immediately after the filling period. Upon termination of the experiment, upper and lower halves of the bladder were also processed histologically. The transurethral catheter results showed that the estrogen and raloxifene-treated animals had significantly higher pressures in responses to rapid stretch whereas the histological analysis showed that the thickness of the epithelial layer, collagen content, and muscle bundles were significantly increased by estrogen and raloxifene treatment.^[10]^

In a study to assess the effect of tamoxifen on periurethral vessels by Doppler velocimetry examination, 21 postmenopausal women with breast cancer in various stages received 20 mg/d tamoxifen for 5 months. The results showed that tamoxifen has an estrogenic action on periurethral blood vessels, decreasing their resistance and increasing their numbers.^[11]^ Thus, it can be speculated that this effect results in improved blood supply to the periurethral muscles which will become more developed and reduce incontinence.

Given all these in vitro, in vivo, and translational experiments, it can be postulated that estrogen and selective estrogen receptor modulators (SERMs) reduce urinary incontinence by different mechanisms. The clinical relevance of these findings is not clear and further investigation is necessary. Indeed, it is known that there is a bidirectional effect of SERMs depending on dosage.^[12]^ A potential explanation is that SERMs at different concentrations may change the gene expression of estrogen receptor (ER) or the ratio of ERa/ERb or the expression of cofactors regulating the receptor activation and downward signal transduction.

In one of the studies, early breast cancer patients (age range, 55–70; mean age, 62.7 ± 4.1) suffering from urinary incontinence after the adjuvant endocrine therapy (estrogen, tamoxifen, and aromatase inhibitors) were asked to fill out the IIQ-7 and UDI-6 questionnaires, and the results were evaluated cross-sectionally. In this study, the difference between urinary incontinence symptoms was statistically insignificant between aromatase inhibitor- or tamoxifen-treated breast cancer patients and control subjects.^[13]^

Another study was done to analyze the changes in frequency and severity of menopausal symptoms in breast cancer patients receiving tamoxifen or aromatase inhibitors. A total of 181 postmenopausal breast cancer patients on endocrine treatment were included in this prospective study. A menopause symptom questionnaire covering vasomotor, atrophic, psychological, cognitive, and urinary symptoms was filled in at baseline, and after 1 and 3 months of therapy. At the end of the study, results showed that for urinary problems no significant changes were seen from baseline to after 1 and 3 months of therapy. The results suggest that tamoxifen does not cause urinary incontinence.^[14]^

In another study, 57 healthy postmenopausal women on raloxifene, tamoxifen, estrogen, and placebo were evaluated for urinary incontinence by the cotton swab test after 20 weeks of treatment. Incontinence was seen in 33% of patients given tamoxifen/raloxifene whereas 18% was seen in the control group. This difference was not statistically significant.^[15]^

Another study, which investigated tamoxifen-related symptoms in 803 breast cancer patients, did not find a statistically significant relationship between the use of tamoxifen and complaints of urinary incontinence.^[2]^

Several clinical studies support the view that the use of estrogen is associated with an increased urinary incontinence complaint.^[16,17]^ Many studies have investigated the effect of raloxifene on urinary incontinence, but they did not demonstrate a significant association between the two.^[15–19]^ So far only 1 study demonstrated that use of raloxifene reduces the incidence of pelvic floor surgery and urinary incontinence.^[20]^ Finally, some studies have shown that levormeloxifene causes a dose-dependent increase in urinary incontinence.^[21,22]^

Whereas some clinical studies show that estrogens cause an increase in incontinence,^[16,17]^ preclinical studies and more clinical studies of both estrogen and SERMs are needed to support this notion. Even though this hypothesis is supported by clinical reports on the use of levormeloxifene,^[21,22]^ the fact that many studies demonstrate that raloxifene has no significant effect on urinary incontinence causes a contradiction.^[15–19]^ In addition, despite the fact that numerous small-scale clinical studies have been done to evaluate the effect of tamoxifen on incontinence, none has proven that a direct relationship exists between the two.^[2,13–15]^

## 4 Conclusion

In conclusion, based on the experience gained from patients admitted to our clinic with this problem, we suggest that clinicians faced with issue should temporarily stop usage of the drug once the complaint of urinary incontinence arises. It is unclear as to whether the results seen in these clinical cases are generalizable to a large population; so more data from long-term prospective studies seems warranted to confirm our findings and also to resolve the contradiction seen between the studies aforementioned.
